# Fabrication of ZnO and TiO_2_ Nanotubes via Flexible Electro-Spun Nanofibers for Photocatalytic Applications

**DOI:** 10.3390/nano11051305

**Published:** 2021-05-15

**Authors:** Monica Enculescu, Andreea Costas, Alexandru Evanghelidis, Ionut Enculescu

**Affiliations:** Laboratory of Multifunctional Materials and Structures, National Institute of Materials Physics, Atomiştilor, 405 A, RO-077125 Magurele, Romania; andreea.costas@infim.ro (A.C.); alex.evanghelidis@infim.ro (A.E.); encu@infim.ro (I.E.)

**Keywords:** ZnO nanotubes, TiO_2_ nanotubes, electrospinning, magnetron sputtering, photocatalysis, solar light, optoelectronic properties, electron microscopy

## Abstract

Web-like architectures of ZnO and TiO_2_ nanotubes were fabricated based on a three-step process of templating polymer nanofibers produced by electrospinning (step 1). The electrospun polymer nanofibers were covered by radio-frequency magnetron sputtering with thin layers of semiconducting materials (step 2), with FESEM observations proving uniform deposits over their entire surface. ZnO or TiO_2_ nanotubes were obtained by subsequent calcination (step 3). XRD measurements proved that the nanotubes were of a single crystalline phase (wurtzite for ZnO and anatase for TiO_2_) and that no other crystalline phases appeared. No other elements were present in the composition of the nanotubes, confirmed by EDX measurements. Reflectance spectra and Tauc plots of Kubelka–Munk functions revealed that the band gaps of the nanotubes were lower than those of the bulk materials (3.05 eV for ZnO and 3.16 eV for TiO_2_). Photocatalytic performances for the degradation of Rhodamine B showed a large degradation efficiency, even for small quantities of nanotubes (0.5 mg/10 mL dye solution): ~55% for ZnO, and ~95% for TiO_2_.

## 1. Introduction

In recent years, one-dimensional nanostructures (nanowires, nanoneedles, nanotubes, nanofibers, nanobelts, etc.) have been in the spotlight of worldwide research due to their remarkable physical and chemical properties induced by the quantum confinement effect of their morphology [1,2]. Among them, nanotubes have drawn considerable attention due to their high surface area, provided by their inner and outer surfaces, making them the perfect candidates for applications in sensors [3], photocatalysis [4], photoelectrochemical water splitting [5], solar cells [6], lithium-ion batteries [7], etc. Until now, semiconductor nanotubes were obtained by various preparation routes, such as electrodeposition [8], hydrothermal [9], precipitation [10], lithographic techniques [11], or by electrospinning associated with other techniques [12,13,14,15]. Among the semiconducting materials, zinc oxide (ZnO) and titanium dioxide (TiO_2_) are n-type semiconductors characterized by a wide band gap (3.37 eV for ZnO and 3.2 eV for TiO_2_), transparency, inherent chemical stability, biocompatibility, and good catalytic activity for contaminant degradation [16,17]. They can be easily prepared in different morphologies (nanowires, nanofibers, nanoflowers, nanotubes, nanorods, etc.) by various facile and low-cost methods [8,10,18,19,20,21,22,23,24,25], making them adequate for a broad range of applications, such as photovoltaics [26], electronic devices [27], photocatalysis [28], water purification [29], dye-sensitized solar cells [30], sensors [31], and renewable energy [32]. 

Electrospinning is a high throughput, cost-effective, and versatile technology used at a large scale to fabricate nanofibers on large surfaces by applying an electrostatic force, using different polymers (polyacrylonitrile, polyvinylpyrrolidone, polyamide, polysulfone, polyethylene glycol, polyester carbonate, polymethyl methacrylate, etc.) or polymer/inorganic hybrid nanocomposites [33]. Radio-frequency (RF) magnetron sputtering is a facile, low-cost deposition technique used to obtain stoichiometric (metallic, semiconductor, or insulator) thin films with a high reproducibility on large surfaces [34].

Up until now, ZnO and TiO_2_ nanotubes were fabricated using distinct approaches, such as, electrodeposition [8,35], hydrothermal [9,36], precipitation [10,37], electrospinning combined with calcination [38,39], and atomic layer deposition [13,40]. To our knowledge, there are only a few reports on the preparation of ZnO nanotubes obtained using electrospinning combined with RF magnetron sputtering and a calcination step [41,42,43]. Nevertheless, the tailoring of the physico-chemical properties of ZnO and TiO_2_ nanotubes provides room for new research.

Herein, we report on the preparation of ZnO and TiO_2_ nanotubes by combining two highly efficient, large-scale methods: electrospinning and RF magnetron sputtering, followed by a calcination step. Hence, we used electrospun PMMA nanofibers as a sacrificial template in the synthesis of nanotubes. The morphological, structural, compositional, and optical properties of the ZnO and TiO_2_ nanotubes were evaluated and discussed in depth. Moreover, the photocatalytic properties of the nanotubes were investigated in order to assess their potential use in applications, such as water splitting and water purification.

## 2. Materials and Methods

### 2.1. Fabrication of ZnO and TiO_2_ Nanotubes

#### 2.1.1. Step 1. Electrospinning of PMMA Fibers

All chemical reagents were acquired from Merck (Darmstadt, Germany) and used without further purification. In order to obtain polymer fibers, we prepared solutions of 10 wt% poly(methyl methacrylate) (PMMA) with Mw = 300,000, with a chemical formula of (C_5_O_2_H_8_)_n_. Dimethylformamide (DMF) was used as the solvent.

To obtain the freestanding polymer fiber webs, large (10 × 10 cm^2^) square copper frames were fabricated and used as collectors in an otherwise classical electrospinning setup, interposed between the syringe needle spinneret and a 20 × 20 cm^2^ aluminum plate acting as a grounded electrode. The distance between the copper frame collectors and the aluminum plate and spinneret was approximately 6 cm and 15 cm, respectively. The 10% (*m/v*) PMMA/DMF solution was fed using a New Era Pump Systems (Farmingdale, NY, USA) syringe pump to a 0.8 mm diameter blunt needle at a 0.5 mL/h rate, with a 15 kV voltage applied to the needle using a Spellman (West Sussex, UK) SL300 HV source. Collection time was 60 min for each copper frame.

#### 2.1.2. Step 2. RF Magnetron Sputtering of ZnO and TiO_2_ Layers

After the electrospinning process, the copper frames containing the PMMA nanofibers were coated on both sides with a semiconducting thin layer (ZnO or TiO_2_) by RF magnetron sputtering employing Tectra GmbH Physikalische Instrumente equipment (Frankfurt, Germany). In the deposition process, a zinc oxide target (ZnO) or a titanium dioxide (TiO_2_) target (acquired from Kurt J. Lesker Company Ltd. Hastings, UK) with a diameter of 2 inches and a thickness of 0.125 inches was used. In the case of ZnO, the following parameters were applied: the working gas was an Ar atmosphere with a purity of 99.999%, the pressure in the deposition chamber was 5.4 × 10^−3^ mbar, the RF power applied on the magnetron was 100 W, and the deposition time was 2 h for each side. In the case of TiO_2_, the following parameters were used: the working gas was an Ar atmosphere, the pressure in the deposition chamber was 4 × 10^−3^ mbar, the RF power applied on the magnetron was 200 W, and the deposition time was 3 h for each side. 

#### 2.1.3. Step 3. Calcination of ZnO and TiO_2_ Nanotubes

**Figure 1:**
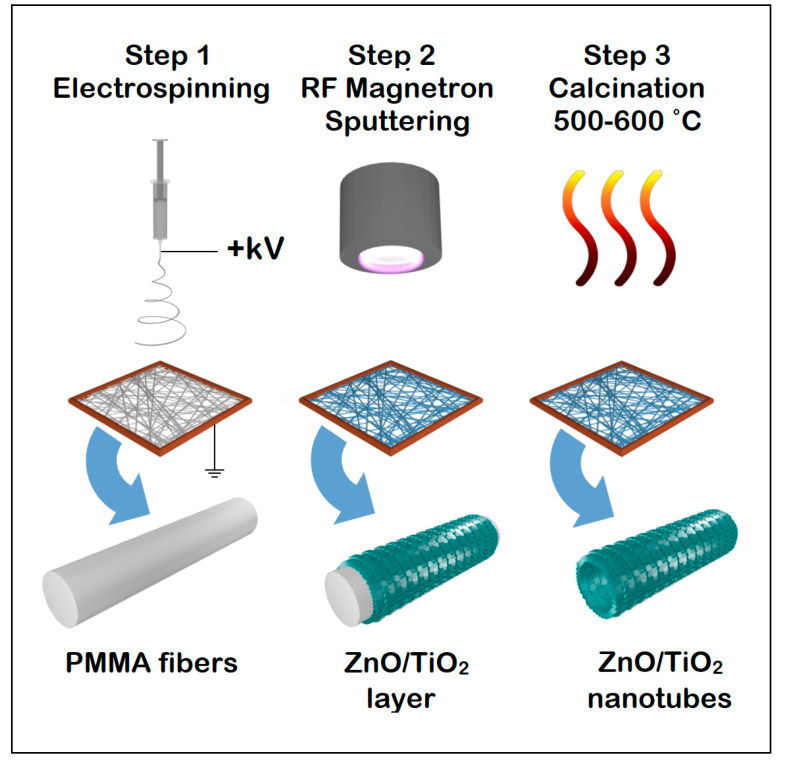
Scheme of the fabrication process of ZnO and TiO_2_ nanotubes.

Subsequently, the PMMA nanofibers covered on both sides with either ZnO or TiO_2_ films were transferred onto Si/SiO_2_ substrates and calcinated using a convection oven from Nabertherm GmbH (Lilienthal, Germany). Hence, the calcination process was performed at 500 °C for ZnO and 600 °C for TiO_2_ for 12 h in the air at ambient pressure. Afterward, 3D web-like networks of ZnO nanotubes (ZnO NT) and TiO_2_ nanotubes (TiO_2_ NT) were obtained, the PMMA being completely burnt out during the calcination process (Figure 1). 

### 2.2. Characterization of ZnO and TiO_2_ Nanotubes

The morphological properties of the ZnO and TiO_2_ nanotube networks were investigated with a Zeiss (Oberkochen, Germany) Merlin Compact field emission scanning electron microscope (FESEM) working in high vacuum (HV), from 0.2 to 30 kV, and equipped with a Zr/W emitter, InLens, and SE2 detectors. 

The elemental composition of the samples was evaluated using a Zeiss (Oberkochen, Germany) Evo 50 XVP scanning electron microscope (SEM) equipped with a Bruker QUANTAX 200 energy dispersive X-ray spectrometer (EDS) with energy resolution < 129 eV at Mn-Ka and Peltier cooling. 

The structural properties of the nanotubes were analyzed with an X-ray diffractometer, XRD AXS D8 Advance instrument with Cu Kα radiation, λ = 0.154 nm (Bruker, Bremen, Germany). Diffraction data were collected from 20° to 65° (2θ).

The optical properties were investigated by reflectance and photoluminescence. The reflectance spectra of the nanotubes were recorded with a Perkin-Elmer (Rodgau, Germany) Lambda 45 spectrometer using an integrating sphere. The photoluminescence excited at λexc = 350 nm and was evaluated using an FL 920 Edinburgh Instruments (Livingston, UK) spectrometer with a 450 W Xe lamp excitation and double monochromators on both excitation and emission, respectively.

The photocatalytic properties of ZnO and TiO_2_ nanotubes were assessed by monitoring the photodegradation of the Rhodamine B (RhB) during the irradiation with solar light. Approximately 0.5 mg of either ZnO or TiO_2_ nanotubes deposited on Si substrates were immersed in a beaker containing 10 mL of RhB aqueous solution (10^−5^ M). Deionized water was obtained using a Millipore system. During the optical measurements, the dye solutions were kept in the dark. Irradiation with white light was performed for 600 min using an SF300-A Small Collimated Beam Solar Simulator (Sciencetech, London, ON, Canada) equipped with an Air Mass AM1.5G Filter (spot size: 25 mm diameter at one Sun) and an integrated electrical shutter with a controller and a Xe lamp (300 W). The samples were positioned 10 cm from the source. During the irradiation, at different time intervals, the optical absorbance spectra of the samples at λ = 400–700 nm were measured using a UV–Vis–NIR CARY 5000 (Varian, Agilent Technologies Deutschland GmbH, Waldbronn, Germany) spectrophotometer provided with a quartz cell with a light path of 10 mm. The characteristic absorption band of RhB, peaking at ~554 nm, was monitored, and its photodegradation was evaluated.

## 3. Results and Discussion

After the removal by calcination of the organic core template formed by the electrospun PMMA nanofibers, the ZnO and TiO_2_ nanotubes were characterized from the morphological, structural, compositional, and optical points of view. 

### 3.1. Morphological Properties

**Figure 2:**
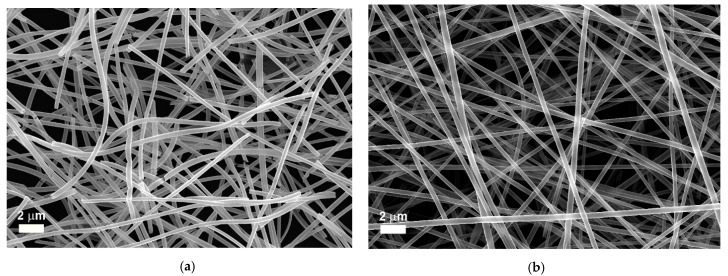

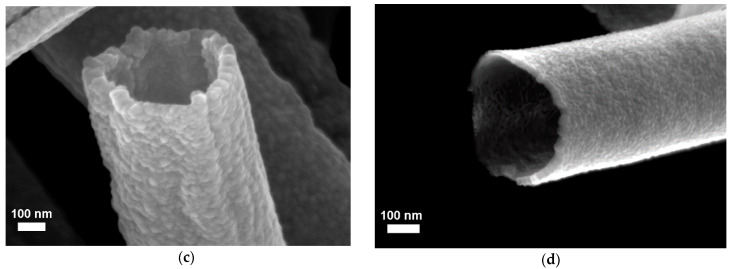
FESEM images of ZnO nanotubes (**a**,**c**) and TiO_2_ nanotubes (**b**,**d**) at different magnifications.

Figure 2 presents high-resolution electron microscopy images of ZnO nanotubes (Figure 2a,c) and TiO_2_ nanotubes (Figure 2b,d). The network architecture provided by the as-prepared electrospun polymeric fibers is further preserved by the nanotubes’ web, as observed in the lower magnification FESEM images (Figure 2a,b). From the point of view of duration, in the conditions presented in the experimental part, the efficiency of ZnO deposition vs. time was higher, whereas, in the case of TiO_2_, the sputtering process had a smaller efficiency. Thus, the sputtering in the case of ZnO had, as a result, a thicker layer of semiconductor material deposited on the surface of the polymer fibers. The higher magnification FESEM images of the nanotubes (Figure 2c,d) revealed that the ZnO nanotubes had an average wall thickness of ~50 nm, whereas the TiO_2_ nanotubes had an average wall thickness of ~35 nm.

The semiconducting layers deposited on the nanofibers are continuous, as observed in the higher magnification FESEM images presented in Figure 2c,d, and have granular morphology for both ZnO and TiO_2_ nanotubes. The coverings with sputtered materials were of uniform thickness all around the surface of the polymeric fibers, as demonstrated by the FESEM images. Because the ZnO nanotubes’ walls are thicker than the walls of the TiO_2_ nanotubes (ZnO nanotubes have a wall thickness of ~50 nm, whereas the TiO_2_ nanotubes have a wall thickness of ~35 nm (or less)), and due to the fact that the density of ZnO (~5.6 g/cm^3^) is larger than the density of anatase TiO_2_ (~3.8 g/cm^3^), the web-like architecture of TiO_2_ nanotubes has a higher active surface. Therefore, the same amount of nanotubes (~0.5 mg) leads to a larger number of nanotubes for TiO_2_ than for ZnO. Although the diameters of the polymeric fibers are similar, the higher active surface provided by the TiO_2_ nanotubes may drastically influence their properties, increasing the photocatalytic activity of those nanostructures. 

### 3.2. Structural and Compositional Properties

#### 3.2.1. XRD Measurements

**Figure 3:**
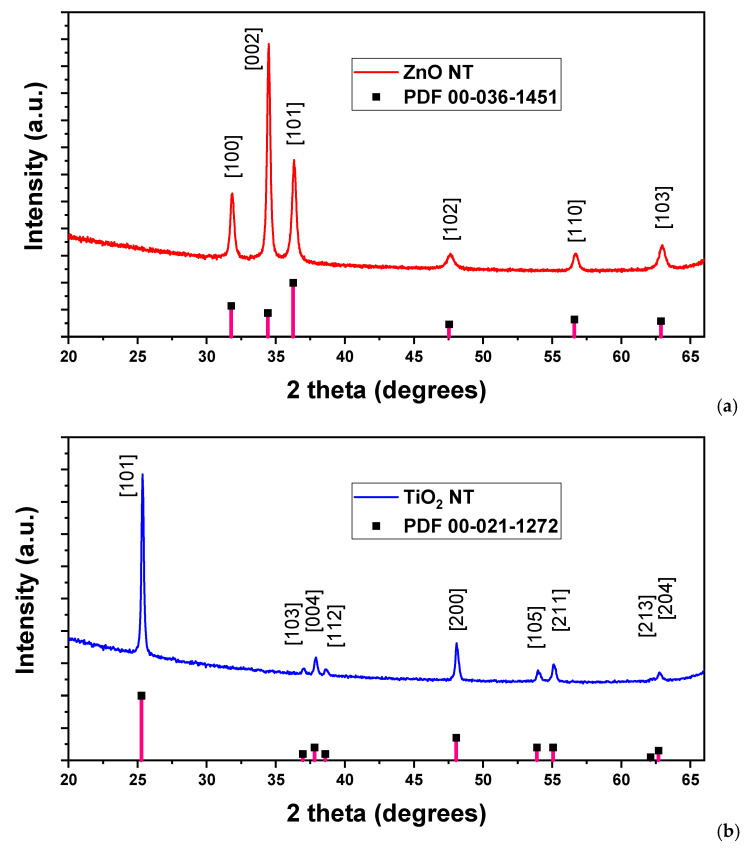
Diffractograms of (**a**) ZnO nanotubes and (**b**) TiO_2_ nanotubes, obtained after sputtering and subsequent calcination.

The structural characterization of the ZnO and TiO_2_ nanotubes fabricated via electrospinning was performed by XRD analysis, as presented in Figure 3. Thus, the diffractogram of ZnO nanotubes obtained after calcination (Figure 3a) exhibits peaks at 2θ: 31.85°, 34.49°, 36.34°, 47.66°, 56.71°, and 62.93°, which can be assigned to (100), (002), (101), (102), (110), and (103) planes of the hexagonal wurtzite ZnO structure, as confirmed by the lines of the ICDD powder diffraction file 00-035-1451, shown in the graph for comparison. The XRD pattern of the TiO_2_ nanotube sample shows only peaks attributed to the anatase form of TiO_2_, corresponding to the (101), (103), (004), (112), (200), (105), (211), (213), and (204) planes of the tetragonal structure, as validated by the lines of the ICDD powder diffraction file 00-021-1272. The peaks present in the XRD pattern of the TiO_2_ nanotubes sample are observed in Figure 3b and appear at the following values of 2θ: 25.36°, 36.99°, 37.87°, 38.64, 48.05°, 53.95°, 55.13°, 62.10°, and 62.76°, respectively. 

In order to better observe the results from the XRD measurements, Table 1 presents the XRD data for ZnO and TiO_2_ nanotubes.

It has to be remarked that all peaks appearing in the ICDD patterns are visible in the ZnO nanotubes and TiO_2_ nanotubes samples’ diffractograms. Moreover, no additional peaks were observed in the XRD patterns of the ZnO or TiO_2_ nanotubes, confirming that the synthesized nanotubes were of a single crystalline phase and that no other additional crystalline structures were obtained after the calcination step.

Further analysis of the XRD results was performed. Thus, the mean sizes of the grains in the single-phase crystalline nanotubes were estimated from the full width at half maximum (FWHM) and Debye–Scherrer formula according to the equation D = 0.9λ/βcos θ, where 0.9 is the shape factor, λ = 0.154 nm is the wavelength of the incident CuKα radiation, β represents full-width at half maximum of the respective peak in radians, and θ is the Bragg diffraction angle. 

The mean size of the grains was calculated using the full width at half maximum (FWHM) of the most intense peaks in the diffractograms and was found to be ~30 nm in the ZnO nanotubes and ~35 nm in the TiO_2_ nanotubes from this Debye–Scherrer equation.

#### 3.2.2. EDX Analysis

**Figure 4:**
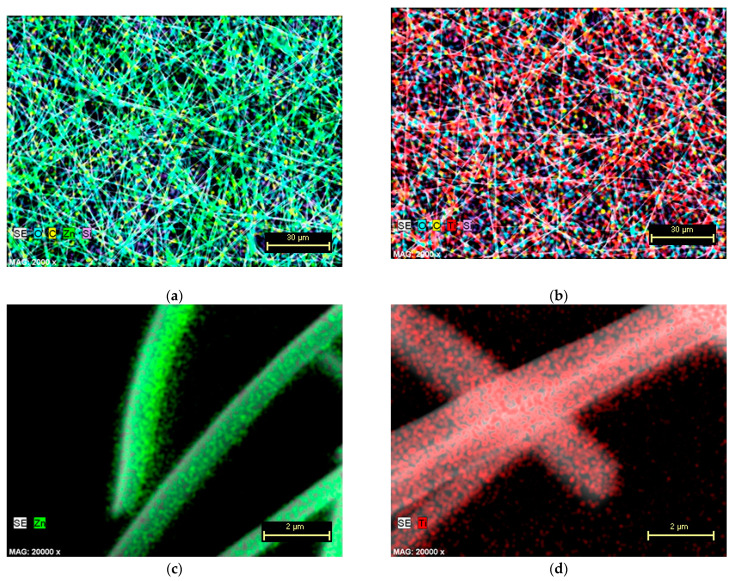
EDX maps at different magnifications showing the uniform distribution of the chemical elements in the (**a**,**c**) ZnO nanotubes and (**b**,**d**) TiO_2_ nanotubes after the removal of PMMA electrospun template by calcination.

The uniformity of the elemental composition of the deposited nanotubes, after the calcination step, was evaluated using the EDX mapping. Thus, the elemental maps of ZnO nanotubes (Figure 4a,c) and TiO_2_ nanotubes (Figure 4b,d) confirm that the semiconducting material is uniformly distributed, and the network replication of the electrospun nanofibers has a uniform composition.

### 3.3. Optical Properties

**Figure 5:**
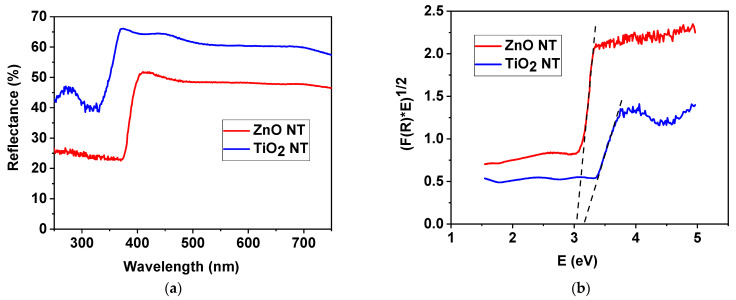

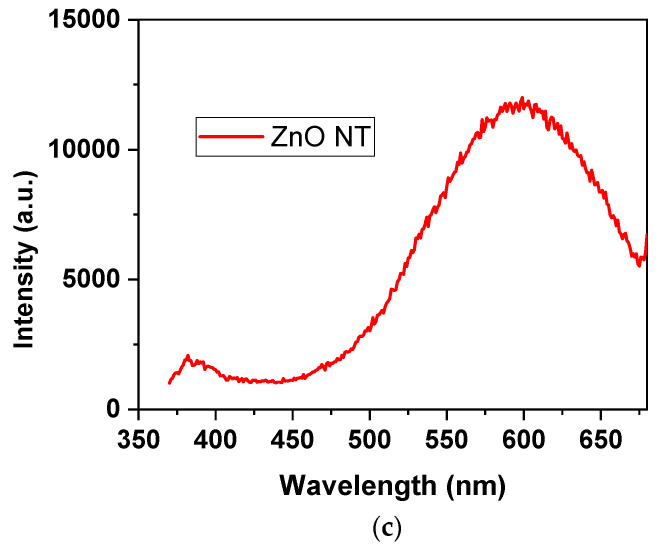
(**a**) Reflectance spectra of ZnO and TiO_2_ nanotubes; (**b**) Tauc’s plots of transformed Kubelka–Munk functions used to estimate the band gap energies of the samples; (**c**) photoluminescence spectrum of ZnO nanotubes excited with 350 nm wavelength.

UV–vis measurements were used in order to evaluate the optical properties of the investigated samples. The UV–vis band gap energies for the samples were determined from the room temperature reflectance (R) spectra. The reflectance spectra of the ZnO and TiO_2_ nanotubes deposited on Si substrates are presented in Figure 5a. It can be observed that, for both types of nanotubes, the reflectance values present slopes that have different angles at wavelengths between 300 nm and 400 nm. 

In order to determine the band gap values of the samples, we calculated the Kubelka–Munk function F(R) = [(1 − R)^2^]/2R, where R is the diffuse reflectance.

Tauc’s plots (F(R) × hν)^n^ vs. hν), where hν is the photon energy and n = ½ for direct band gap semiconductors, were used in order to determine the band gap energies for the samples. 

Thus, the band gap energies were estimated from the intersections of the tangents to the slopes in the curves with the photon energy axis, as illustrated in Figure 5b. The nanotubes show values of Eg of about 3.04 eV for ZnO and 3.16 eV for TiO_2_. The band gap of the ZnO nanotubes is consistent with studies presented in the literature that underline that the effective band gap of ZnO nanostructures is smaller than the value for the bulk material of 3.37 eV [16]. On the other hand, a very small shift to lower values of the energies is observed for the band gap of TiO_2_ nanotubes when compared with the band gap of bulk TiO_2_ (3.2 eV for anatase) [17]. Both values calculated for the band gap energies suggest that the nanotubes will present photocatalytic properties at wavelengths higher than those corresponding to UV range. 

The shift in the band gap energy’s value is either an effect due to the modification of the structural properties of the semiconductor or an effect caused by doping the material. It is well known that the band gap values for semiconductors should increase with a decrease in size because of the modification of the spacing of the electronic levels. However, there can be situations when, even if the semiconductor is nanostructured, a decrease in band gap energy value may be observed. Usually, the narrowing of the band gap is due to the impurities present in the material. However, a decrease in the band gap can be explained based on the presence of a mixture of oxidation states similar to the ones induced by the doping of the material. The XRD confirmed that there is one single crystalline phase for the nanotubes; therefore, the decreasing of the bandgap is a result of the oxidation states induced by structural imperfections [44].

The photoluminescence spectrum of the ZnO nanotubes presents two bands, typical for this semiconducting material (Figure 5c). Thus, a weak band may be observed peaking in the ultraviolet region, with a maximum at ~380nm, being related to the band-to-band transitions and appearing due to the recombination of the excitons [35]. The more intense and broad band that may be observed in the visible region, with the maximum at ~595 nm, is related to various defects that are formed in ZnO (vacancies, interstitials, etc.) [35]. 

### 3.4. Photocatalytic Activities

**Figure 6:**
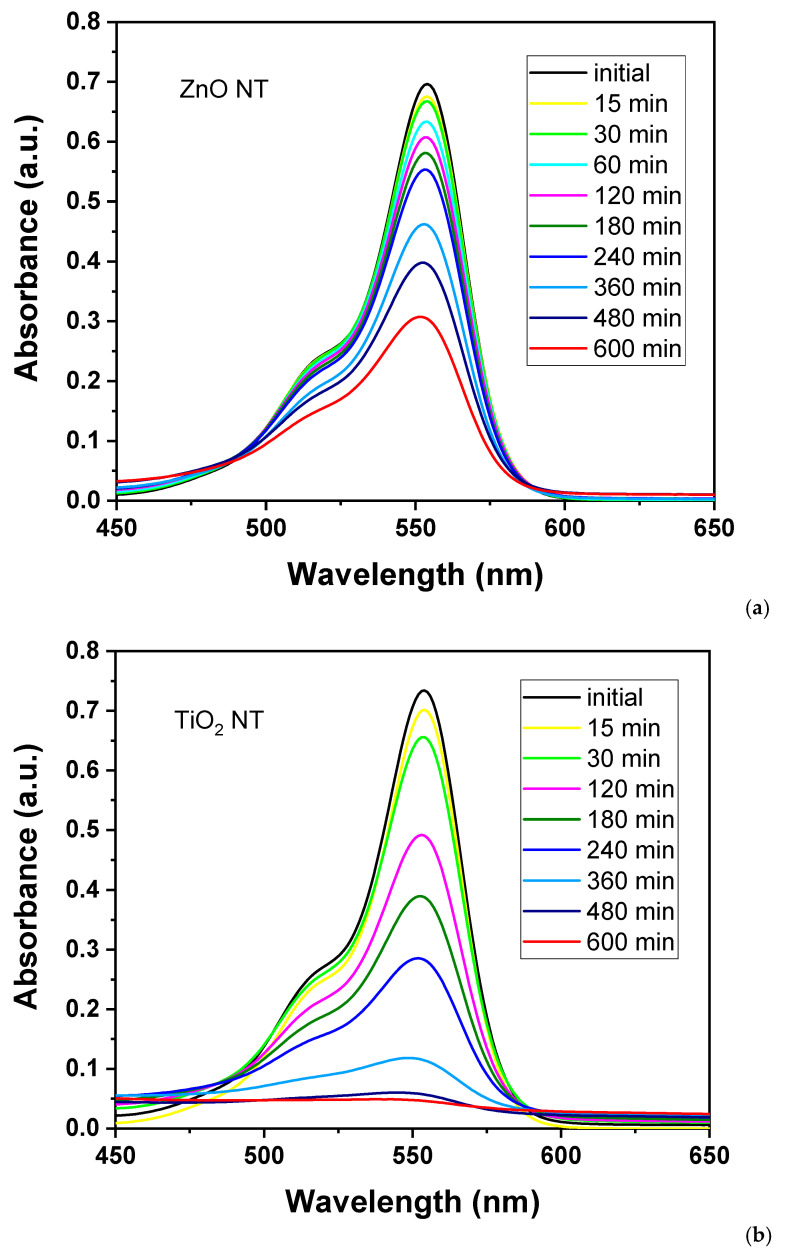
Absorption spectra of RhB solutions after irradiation with white light of a solar simulator for different times in the presence of (**a**) ZnO nanotubes and (**b**) TiO_2_ nanotubes.

The photocatalytic activity of the nanotubes was evaluated by Rhodamine B degradation experiments under irradiation with a solar simulator’s white light employing aqueous solutions in which the nanotubes were immersed. The self-degradation of Rhodamine B under the solar light in the absence of the samples was negligible. In order to evaluate the degradation that occurred when nanotubes were immersed in the dye’s solutions, 3 mL of RhB solution was collected from the beaker at different intervals of time, and the absorption spectra were registered (Figure 6). The irradiation with white light was started immediately after the immersion of the samples. The degradation process was monitored for 600 minutes in order to observe if, for any of the samples, a total degradation of the dye was obtained or until we achieve a saturation threshold. 

During the absorption measurements, the irradiation with the solar simulator was interrupted. The solution was kept in the dark and, after the re-injection in the beaker of the measured amount, the whole solution was homogenized. Comparative with the total time of irradiation, the absorbance measurements were performed quickly, excluding a supplementary degradation due to the spectrometric evaluation.

In order to exclude the influence of secondary processes such as adsorption effects and prove that the degradation of Rhodamine B is not due to adsorption into the nanotubes, ZnO nanotubes were immersed in RhB solution in the dark for a total of 36 h (Figure S1a). After the sample removal, there was no indication that adsorption processes took place (Figure S1b), and the intensity of the absorption band of Rhodamine B after 36 h was similar to the initial intensity (Figure S1c).

As observed in Figure 6, the photocatalytic effect registered for ZnO nanotubes (illustrated in Figure 6a) is less effective than the degradation observed for the TiO_2_ nanotubes sample (illustrated in Figure 6b). 

After increasing the reaction time, the photodegradation rate of the organic dye decreases. In order to observe the kinetics of the degradation, the degradation efficiency was calculated using the following equation:

Degradation efficiency (%) = (C_0_ − C)/C_0_ × 100

where C_0_ is the absorbance value before irradiation at t = 0 min, and C is the absorbance value at particular intervals of time (t = 15 min, 30 min, 60 min, 120 min, etc.).

**Figure 7:**
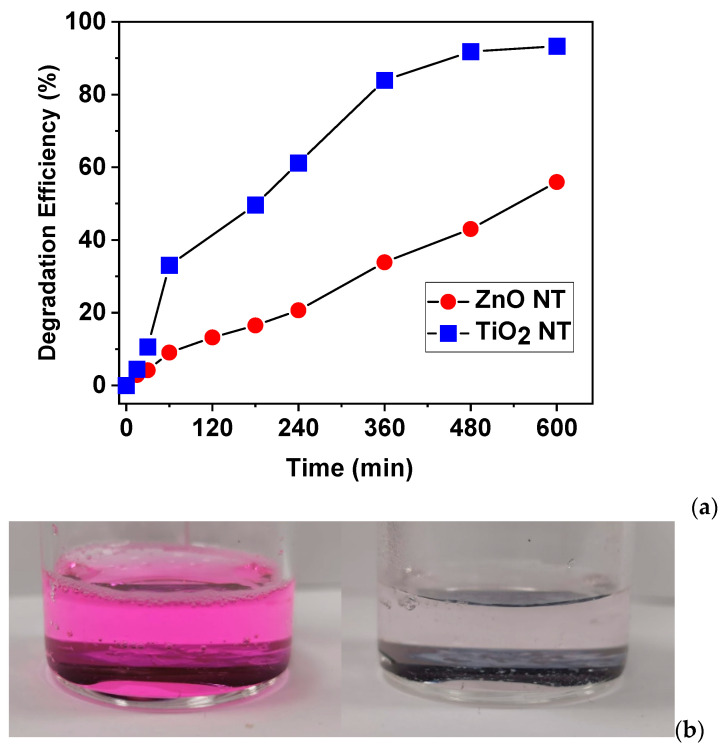
(**a**) Degradation efficiency of ZnO and TiO_2_ nanotubes; (**b**) RhB solution before and after irradiation under solar simulator light.

The plot of degradation efficiency vs. time for both ZnO and TiO_2_ nanotubes is presented in Figure 7a. It was observed that a total bleaching of the RhB solution was obtained for TiO_2_ nanotubes after prolonged irradiation time, as illustrated in Figure 7b. The photocatalytic degradation efficiency is 95% for the TiO_2_ nanotubes and 55% for the ZnO nanotubes, respectively. The degradation efficiency values of the nanotubes are consistent with data previously reported in the literature for ZnO and TiO_2_ nanostructures [28,37,45,46,47].

**Figure 8:**
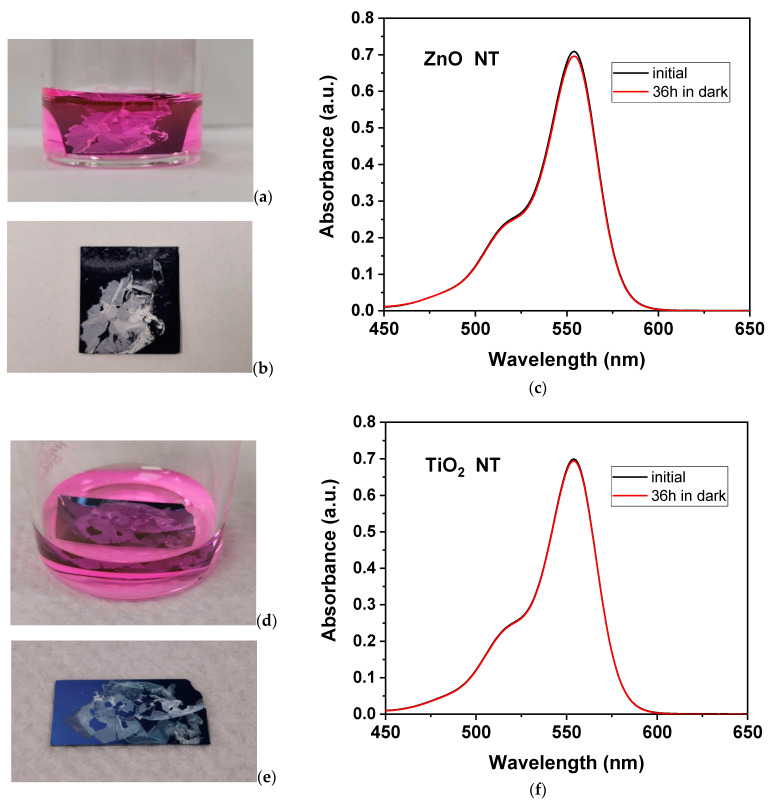
(**a**) ZnO NT inserted in RhB solution; (**b**) sample of ZnO NT on Si substrate after removal from RhB solution in which was kept in dark for 36 h; (**c**) initial and after 36 h spectra of RhB solution in which ZnO NT was immersed; (**d**) TiO_2_ NT inserted in RhB solution; (**e**) sample of TiO_2_ NT on Si substrate after removal from RhB solution in which was kept in dark for 36 h; (**f**) initial and after 36 h spectra of RhB solution in which TiO_2_ NT were immersed.

With the purpose of excluding the influence of secondary processes such as adsorption effects and to prove that the degradation of Rhodamine B is not due to adsorption into the nanotubes, experiments were performed in dark. Thus, ZnO nanotubes were immersed in RhB solution in the dark for a total of 36 h (Figure 8a). After sample removal, there was no indication that adsorption processes took place (Figure 8b), and the intensity of the absorption band of the Rhodamine B band after 36 h was similar to the initial intensity (Figure 8c). Similarly, TiO_2_ nanotubes were immersed in Rhodamine B solution (Figure 8d), and no indication of the adsorption effect was observed after 36 h in the dark (Figure 8e), with no diminishing in the dye’s absorption band (Figure 8f). 

**Figure 9:**
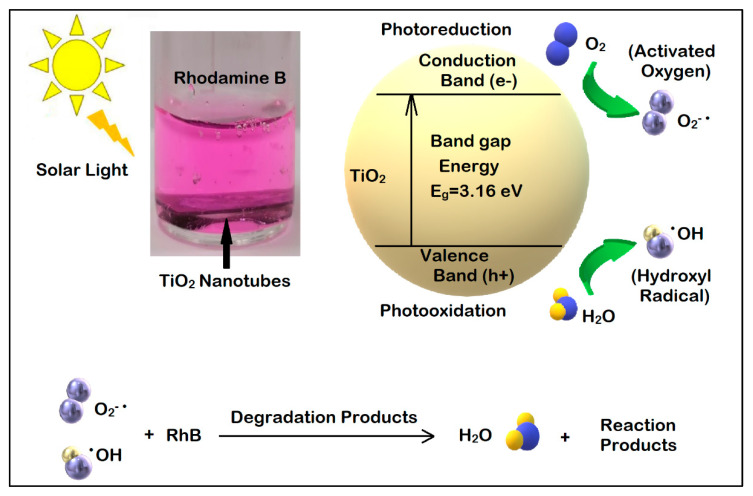
Mechanism of photocatalysis in the presence of the TiO_2_ nanotubes and the reactions for the degradation of RhB.

In order to explain the mechanism of degradation of Rhodamine B in the presence of the nanotubes, the formation of the oxidant agents in the mechanism of degradation of Rhodamine B is presented schematically, using TiO_2_ nanotubes to exemplify the processes. Figure 9 illustrates the types of reactions (oxidative and reduction) that take place in the presence of the metal oxide semiconductor and the photocatalysis process.

In photocatalytic degradation based on semiconducting nanotubes, a photon with an energy higher than the energy of the semiconductor’s band gap activates the electron in the valence band to make the band-to-band transition, producing an electron–hole pair. The charge carriers from the surface of the nanotubes interact with the chemicals in the aqueous solution, degrading them and forming the reaction products. The electron in the conduction band is involved in the reduction reaction of O_2_, while the hole generated in the valence band participates in the oxidation reaction that produces the hydroxyl ion, as illustrated in Figure 9. Both types of oxidant agents may interact with Rhodamine B molecules leading to, after the formation of intermediate products, H_2_O and CO_2_ molecules.

Although the pure ZnO and TiO_2_ have lower degradation efficiencies when compared with doped ZnO and TiO_2_ [48], nanostructuring these types of materials, i.e., reducing their dimensions, leads to larger specific surface areas. The nanotubes offer a doubled area for photocatalytic reactions. Moreover, the granulated morphology further increases that area. This is confirmed by the larger degradation efficiency of the TiO_2_ nanotubes (~95%) compared with one of the ZnO nanotubes (~55%) due to the thinner walls observed by FESEM evaluations in the case of TiO_2_ nanotubes.

The main advantage offered by the ZnO and TiO_2_ nanotubes for their use as photocatalysts for pollutants’ degradation is that they can be easily removed from the solution at the end of the degradation process. Further, the nanotubes can be used either immobilized on a substrate or by insertion in a porous composite.

## 4. Conclusions

A three-step fabrication process was used in order to obtain semiconducting nanotubes for optoelectronic and photocatalytic applications. Polymer fibers produced by electrospinning were covered over their entire surface via RF magnetron sputtering with a thin layer of semiconductor oxide. After being used as templates, the polymer fibers were removed by calcination leading ZnO and TiO_2_ nanotubes to assemble in web-like architectures. The single crystalline phase, wurtzite ZnO, with a band gap of 3.05 eV and intense luminescence, and anatase TiO_2_ with a band gap of 3.16 eV present interesting photocatalytic properties demonstrated by Rhodamine B degradation under irradiation with white light of a solar simulator, having a degradation efficiency of ~55% for ZnO nanotubes and ~95% for TiO_2_ nanotubes. The interesting capabilities of the ZnO and TiO_2_ nanotubes are given by the fact that the photocatalytic process takes place up to the total bleaching of contaminants in solutions, even for a small number of nanotubes, and by the possibility of removal of web-like constructed nanotubes after the degradation process.

## Figures and Tables

**Table 1:** XRD data for ZnO and TiO_2_ nanotubes.

ZnO Nanotubes		TiO_2_ Nanotubes	
Peak Position (2θ °)	hkl	Peak Position (2θ °)	hkl
31.85 34.49 36.34 47.66 56.71 62.93	100 002 101 102 110 103	25.36 36.99 37.87 38.64 48.05 53.95 55.13 62.10 62.76	101 103 004 112 200 105 211 213 204

## Supplementary Materials

The following are available online at https://www.mdpi.com/article/10.3390/nano11051305/s1, Figure S1: (a). ZnO NT inserted in Rhodamine B solution; (b). Sample of ZnO NT on Si substrate after re-moval from Rhodamine B solution in which was kept in dark for 36 h; (c). Initial and after 36 h spectra of Rhodamine B solution.
