# Supplementary material for: Fabrication of ZnO and TiO2 Nanotubes via Flexible Electro-Spun Nanofibers for Photocatalytic Applications
Source: Nanomaterials (Basel). 2021 May 15;11(5):1305. doi: 10.3390/nano11051305 (PMC8156990; doi:10.3390/nano11051305)
Supplement: Supplementary file 1 [file nanomaterials-11-01305-s001.zip › nanomaterials-1190057-supplementary.pdf]

nanomaterials

Supplementary Materials

Fabrication of ZnO and TiO_2 Nanotubes via Flexible Electro-Spun Nanofibers for Photocatalytic Applications

Monica Enculescu *,† Andreea Costas †, Alexandru Evanghelidis and Ionut Enculescu

Laboratory of Multifunctional Materials and Structures, National Institute of Materials Physics, Atomiştilor, 405 A, RO-077125 Magurele, Romania; andreea.costas@infim.ro (A.C.); alex.evanghelidis@infim.ro (A.E.); encu@infim.ro (I.E.)

† Correspondence: authors contributed equally.

* Correspondence: mdatcu@infim.ro

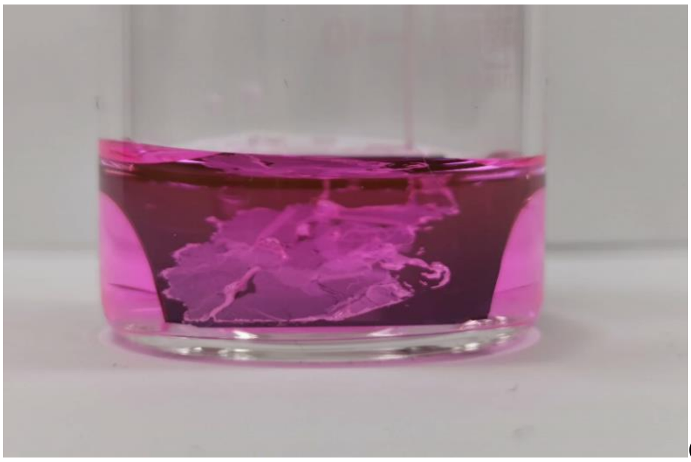

(a)

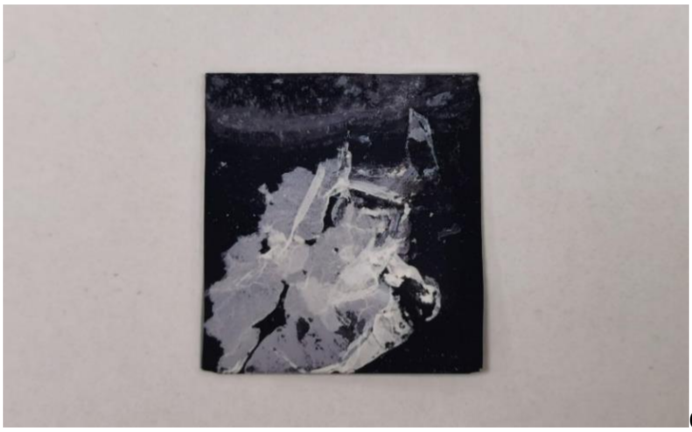

(b)

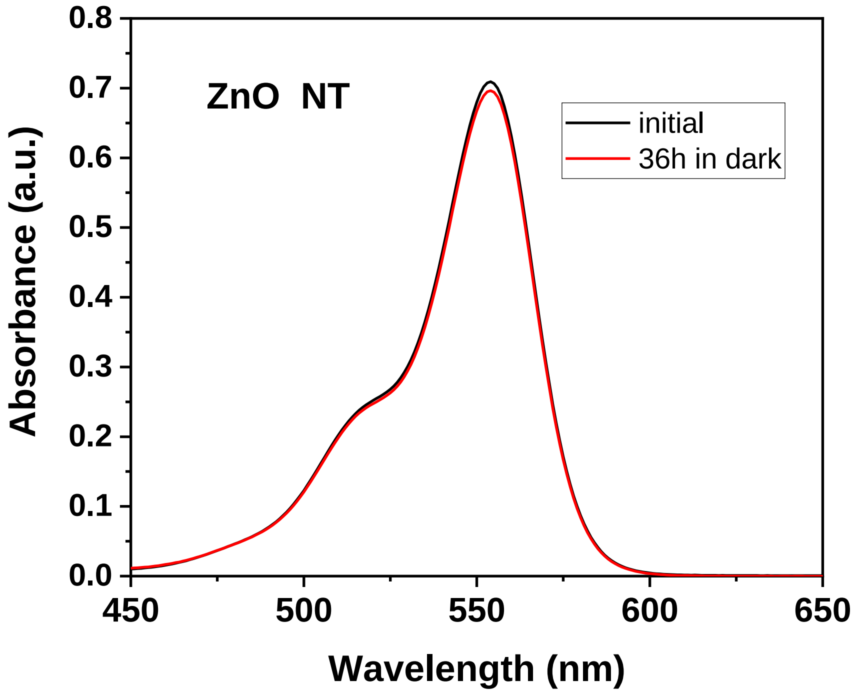

(c)

Figure S1. (a). ZnO NT inserted in Rhodamine B solution; **(b)**. Sample of ZnO NT on Si substrate after removal from Rhodamine B solution in which was kept in dark for 36 h; **(c)**. Initial and after 36 h spectra of Rhodamine B solution.
